# Comparative effectiveness of interventions for managing urological postoperative catheter-related bladder discomfort: a systematic review and network meta-analysis

**DOI:** 10.1186/s12894-023-01195-9

**Published:** 2023-03-03

**Authors:** Jingwen Ren, Ting Yu, Ye Tian, Guangheng Luo

**Affiliations:** 1 Department of Urology, Guizhou Province People’s Hospital, Guiyang, China; 2 Evidence-Based Nursing Center, West China Hospital, Sichuan University/West China School of Nursing, Sichuan University, Chengdu, China

**Keywords:** Catheter-related bladder discomfort, Network meta-analysis, Nefopam

## Abstract

**Background:**

Catheter-related bladder discomfort (CRBD) is a common postoperative bladder pain syndrome. Many drugs and interventions for managing CRBD have been studied, but their comparative effectiveness remains controversial. We made a study to assess the comparative effectiveness of interventions included Ketorolac, Lidocaine, Chlorpheniramine, Gabapentin, Magnesium, Nefopam, Oxycodone, Parecoxib, Solifenacin, Tolterodine, Bupivancaine, Dexmedetomidine, Hyoscine N-butyl bromide, Ketamine, Penile nerve block on urological postoperative CRBD.

**Methods:**

We performed a network meta-analysis via Aggregate Data Drug Inormation System software included 18 studies with 1816 patients and assessed the risk of bias by Cochrane Collaboration tool. The incidence of moderate to severe CRBD at 0, 1, and 6 h after surgery and the incidence severe CRBD at 1 h after surgery were compared.

**Result:**

The number of best rank is 0.48(Nefopam) and 0.22(Nefopam) in the incidence of moderate to severe CRBD at 1 h and incidence severe CRBD at 1 h. More than half of studies at unclear or high risk of bias.

**Conclusion:**

Nefopam reduced the incidence of CRBD and prevented severe events, but limited by the small number of studies for each intervention and heterogeneous patients.

**Supplementary Information:**

The online version contains supplementary material available at 10.1186/s12894-023-01195-9.

## Introduction

Surgical intervention involving urinary catheterization often results in CRBD, turning it into an important clinical entity with an incidence of 47–90% [1]. The symptoms of CRBD usually include a burning sensation in the suprapubic region and penile discomfort during urination. In addition, these complaints can lead to various behavioral responses, including catheter removal, strong vocal responses, and shaking of limbs [1, 2]. As CRBD may exacerbate postoperative pain and reduce the quality of life, it is necessary to investigate drugs or interventions that could reduce the incidence and severity of CRBD.

The characteristics of CRBD are similar to that of overactive bladder (OAB), and clinical symptoms of both these conditions are related to involuntary detrusor contractions [3]. Bladder stimulation and contraction from the catheter are mediated by stimulation of muscarinic receptors. Previous studies reported that muscarinic receptors in the bladder were heterogeneously distributed, with M2 receptors being the dominant ones and M3 receptors less prominent. As activation of M2 receptors causes contraction of detrusor smooth muscle, resulting in dysuria, urgency, frequency, and burning sensation in the suprapubic region [4], the effect of drugs with antimuscarinic activity such as oxybutynin, butylscopolamine, tolterodine, solifenacin was investigated in prevention or treatment of CRBD [5, 6]. Promising results were recently reported for the central alpha2-adrenoceptor agonist dexmedetomidine [7]. Gabapentin and peripheral nerve blocks, including coccygeal nerve block, dorsal penile nerve block, and lidocaine prolocaine cream, have been reported to have a beneficial effect [3, 8, 9]. Anesthetics such as ketamine, sevoflurane, desflurane, and propofol were also used to compare the effects of postoperative CRBD [10]. Although a few accounts or systematic reviews of these drugs and procedures have been published, a small number of positive comparisons of these interventions have been made. Although many studies evaluated the efficacy of drugs with different mechanisms of action for CRBD, little is known about their relative efficacy. Therefore, a direct comparison of the effectiveness of all interventions is needed to review the current evidence and guide further clinical trials.

A NMA is a statistical approach that compares different drugs that have not yet been directly compared by head-to-head randomized controlled trials (RCT) [11]. Based on statistical inference, the relative advantage of any one modality compared to other drugs can be determined, and its relative ranking estimated [12]. Therefore, we performed NMA on managing urological postoperative CRBD, which included 18 recent studies published from 2014 to 2021 and the comparison of the effectiveness of 16 interventions. These comparisons were performed in the Bayesian network model by using ADDIS software. We obtained the comparative effectiveness of these interventions in relation to the incidence and severity of postoperative CRBD (Additional files 1, 2).

## Materials and methods

In the present study, we conducted a network meta-analysis in accordance with the Preferred Reporting Items for Systematic Reviews and Meta-Analyses (PRISMA) guidelines [13, 14]. The present study was registered at PROSPERO (ID: CRD42022313578) (https://www.crd.york.ac.uk/PROSPERO/).

### Eligibility criteria and search strategy

**Fig. 1:**
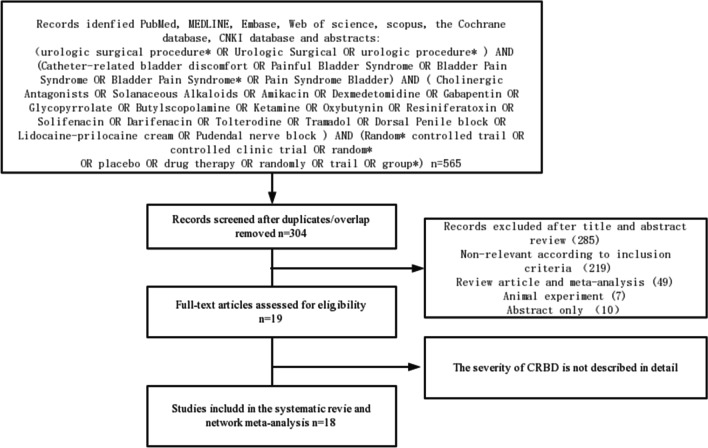
Preferred reporting items for systematic reviews and meta-analyses flow chart depicting the studies and abstracts included in the part of network meta-analysis of managing CRBD

PubMed, Medline, Embase, Web Of Science, Scopus, Cochrane database, China National Knowledge Internet (CNKI) database, and recent abstracts were independently searched by two authors to identify all available RCTs addressing the therapy of postoperative CRBD (Fig. 1). Inclusion criteria for relevant RTCs were following: (1) patients aged ≥ 18 years undergoing any types of urological surgeries requiring placement of urinary catheter; (2) at least one drug or intervention with placebo control; (3) report incidence or severity of postoperative CRBD with at least one of the following time frames: 0, 1, and 2 or 6 h postoperatively; (4) the severity of CRBD was described in detail, or the scoring criteria for severity definition were developed. In addition, some RCTs evaluating the effects of interventions in patients with CRBD were also included and analyzed. The aim of all All RCTs was to evaluate the effects of any of the following drugs or interventions, which were preoperatively and/or intraoperatively used: cholinergic antagonists, solanaceous alkaloids, dexmedetomidine, gabapentin, glycopyrrolate, butylscopolamine, ketamine, oxybutynin, solifenacin, darifenacin, tolterodine, tramadol, lidocaine-prilocaine cream, and resiniferatoxin. The penile nerve block was also included as a non-drug treatment because there were two RCTs describing its effect [15, 16].

### Data extraction and management

Two authors independently extracted data using a unified form developed by the authors (Table 1). Any differences were resolved by consensus. In addition, the following information was extracted from each trial: study location, year of publication, number of patients enrolled, inclusion and exclusion criteria, side effects, and distribution of outcome data.

**Table 1:** Number of included studies and enrolled patients according to the individual interventions

Interventions	Studies (n)	Patients (n)	Operation	Published year
Ketorolac	1	65	ULS	2014
Lidocaine	1	66	TURBt	2020
Ketamine	2	82	TURBt	2016
Parecoxib	1	61	TURBt	2018
Solifenacin	1	62	TURBt	2015
Magnesium	1	60	TURBt	2020
Dexmedetomidine	2	79	TURBt	2015–2016
Chlorpheniramine	2	76	TURBt&UL	2021
Nefopam	2	63	TURBt&UL	2018–2021
Penile nerve block	2	126	TURBt&TURP	2017–2021
Gabapentin	1	50	PCNL	2018
Tolterodine	1	50	PCNL	2018
Bupivancaine	1	31	PCNL	2020
Oxycodone	1	46	TURP	2019
Hyoscine N-butyl bromide	1	24	TURP	2017
Placebo	18	875		2014–2021
Total	18	1816		2014–2021

*ULS* urology laparoscopic surgery, *UL* ureteroscopic lithotripsy, *TURBt* Transurethral Resection of Bladder tumors, *TURP* transurethral resection of prostate, *PCNL* percutaneous nephrolithotomy

### Outcome definitions

Moderate and severe CRBD were defined according to the article's description of the severity of CRBD. Severe CRBD was defined as the presence of excruciating pain accompanied by restlessness, self-extraction of a urinary catheter, uncontrolled shaking of limbs, and pain that would not relieve itself on its own. Moderate CRBD was defined based on the following criteria: significant but tolerable pain, pain without agitation, self-relieving pain, and a lower pain score than severe CRBD. The primary endpoint was the incidence of moderate to severe CRBD at 1 h postoperatively because this time point had a larger incidence and greater consistency than other time points in included studies. Secondary endpoints included the incidence of moderate to severe CRBD at 0 and 6 h postoperatively. The incidence of severe CRBD at 1 h after surgery was compared in terms of severity.

### Statistical analysis and risk of bias

**Fig. 2:**
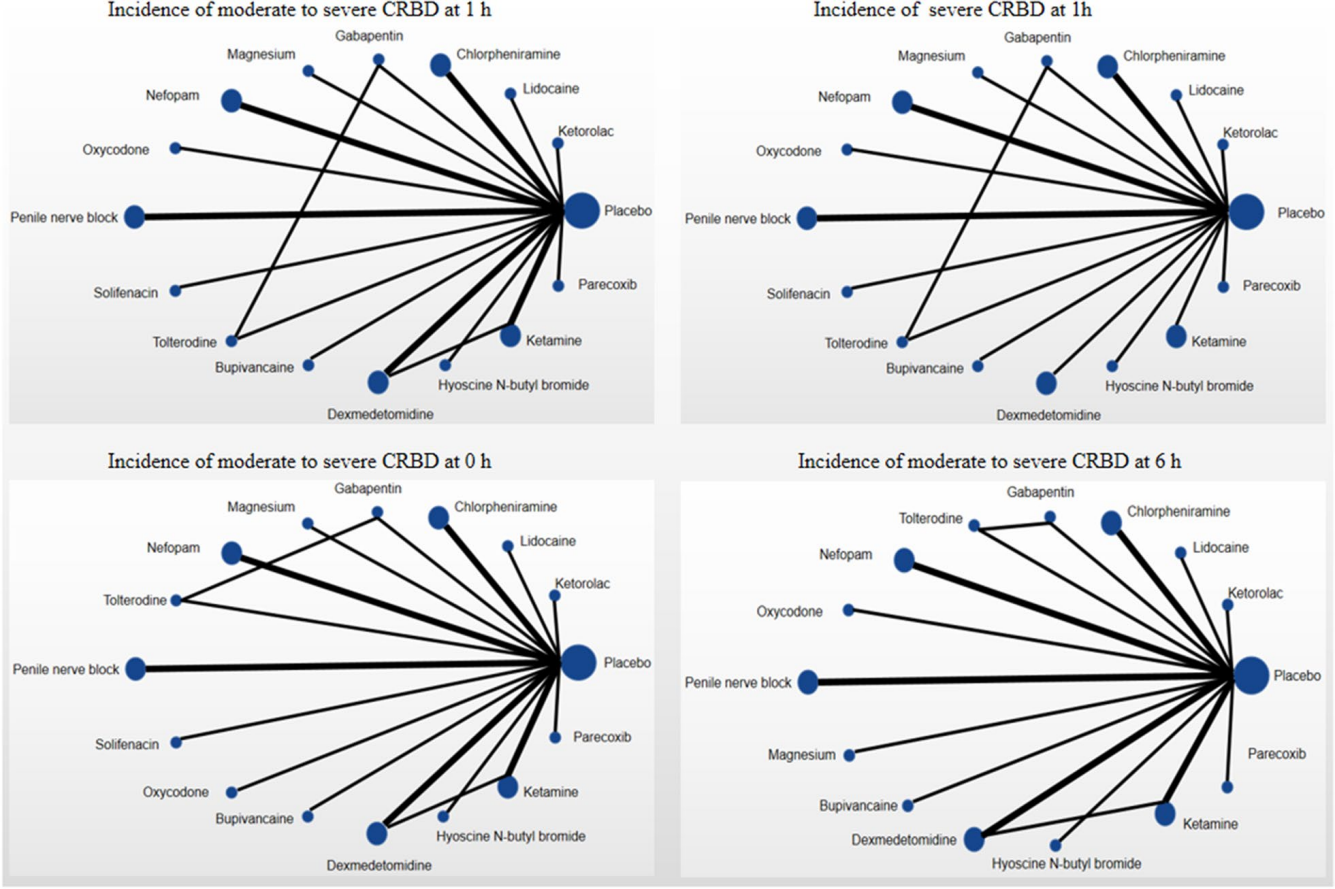
Networking of possible interventions based on our four results. Nodes are weighted according to the number of patients with the corresponding intervention. Edges are weighted according to the number of studies between two connected modes

We designed an NMA using stochastic models and used a Bayesian approach to directly and indirectly compare the efficacy of drugs with different mechanisms of action for postoperative CRBD [17, 18] (Fig. 2). ADDIS1.16.8 software was used for data analysis. We calculated direct and indirect odds ratios (OR) and 95% confidence intervals (95%CI) to analyze the effect on efficacy. The node-splitting model was used to test the consistency between closed-loop studies with both direct evidence and indirect evidence. If there was no significant difference (P > 0.05), the consistency model was used for network meta-analysis; if there was a significant difference (P < 0.05), the source of inconsistency was analyzed. The potential scale reduced factor (PSRF) reflects the convergence; if the PSRF is close to 1, it indicates that the convergence is better, the model analysis results are stable, and the conclusion is highly credible. The result is acceptable if PSRF < 1.02 (Table 2). The risk of bias was evaluated for each study according to the Cochrane Collaboration tool [19]. This tool assesses selection bias, performance bias, attrition bias, detection bias, reporting bias, and other sources of bias.

**Table 2:** The potential scale reduced factor of Bayesian model

Parameter	PSRF
d.placebo.Chlorpheniramine	1.00
d.placebo.Gabapentin	1.00
d.placebo.Ketorolac	1.01
d.placebo.Lidocaine	1.00
d.placebo.Magnesium	1.00
d.placebo.Nefopam	1.00
d.placebo.Oxycodone	1.00
d.placebo.Parecoxib	1.01
d.placebo.Solifenacin	1.00
d.placebo.Tolterodine	1.00
d.placebo.bupivancaine	1.00
d.placebo.dexmedetomidine	1.00
d.placebo.Hyoscine N-butyl bromide	1.00
d.placebo.ketamine	1.00
d.placebo.pudendalnerveblock	1.00
sd.d	1.00

## Results

As shown in Fig. 1, 565 articles were initially screened. Next, 304 overlapping articles were eliminated, and 19 articles were kept after reading their titles and abstracts. Among these, 1 study was eliminated as it was impossible to obtain complete data, and 18 studies were finally included after carefully reading the full text.

**Fig. 3:**
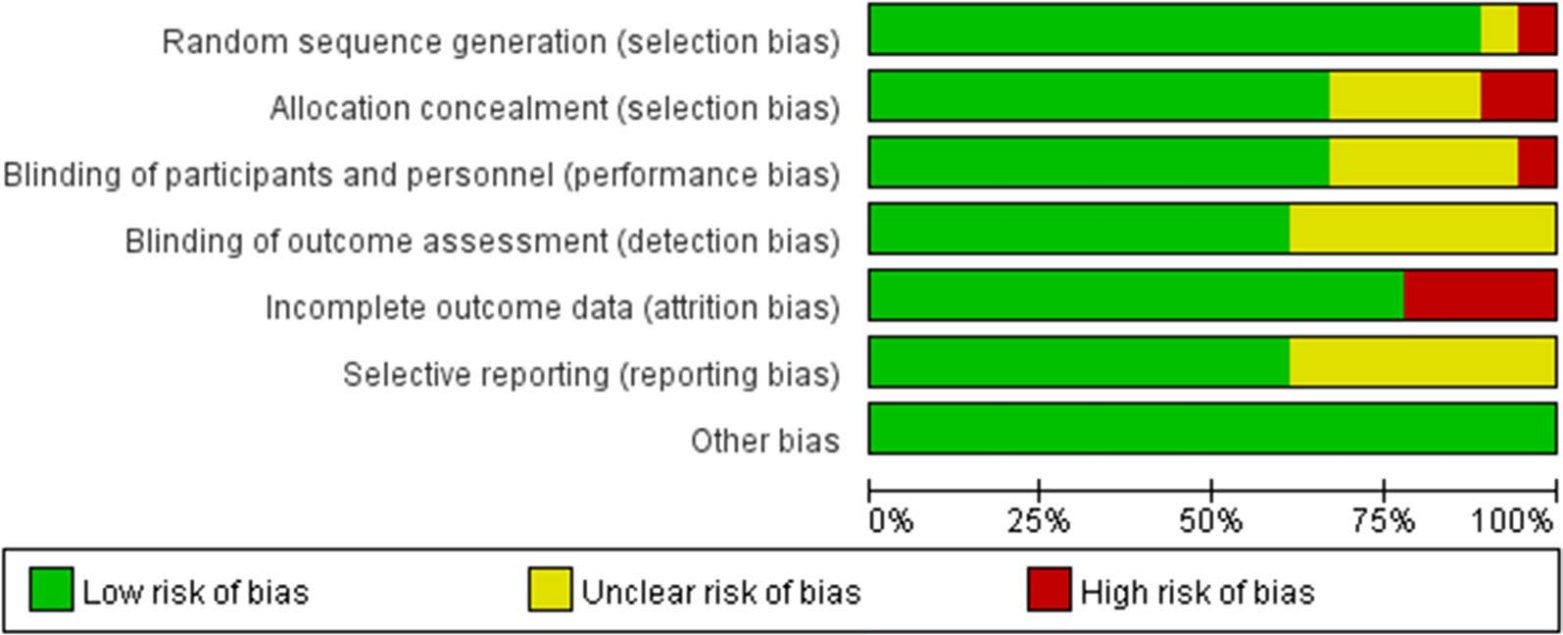
Risk of bias graph: review authors' judgements about each risk of bias item presented as percentages across all included studies according to Cochrane Risk bias assessment tool

Figure 3 shows the risk of bias assessment of included studies based on the Cochrane Risk bias assessment tool. All included studies were reported to be randomized. However, one of these studies did not describe the randomization procedure in enough detail to allow for assessment of risk of bias and thus was considered as having an unclear risk. Another study did not use the right randomization procedure, so it was assessed as having a high risk. Eight studies were evaluated as having a high or unclear risk of bias for blinding of outcomes as authors failed to describe the blinding method, or most outcomes were assessed by self-report scales, thus making them subjective. However, 4 studies were rated as having high risk as they did not provide data about the flow of participants over the study period or due to failure to explain the imbalance in participants between the two groups. Seven studies were rated as having an unclear risk of bias due to a lack of information on the prediction of outcomes at the protocol stage and a lack of clarification on whether they were primary or secondary outcomes. Also, there were no other risks of bias among included studies. The low risk of bias was defined at > 50% in six assessment domains.

**Fig. 4:**
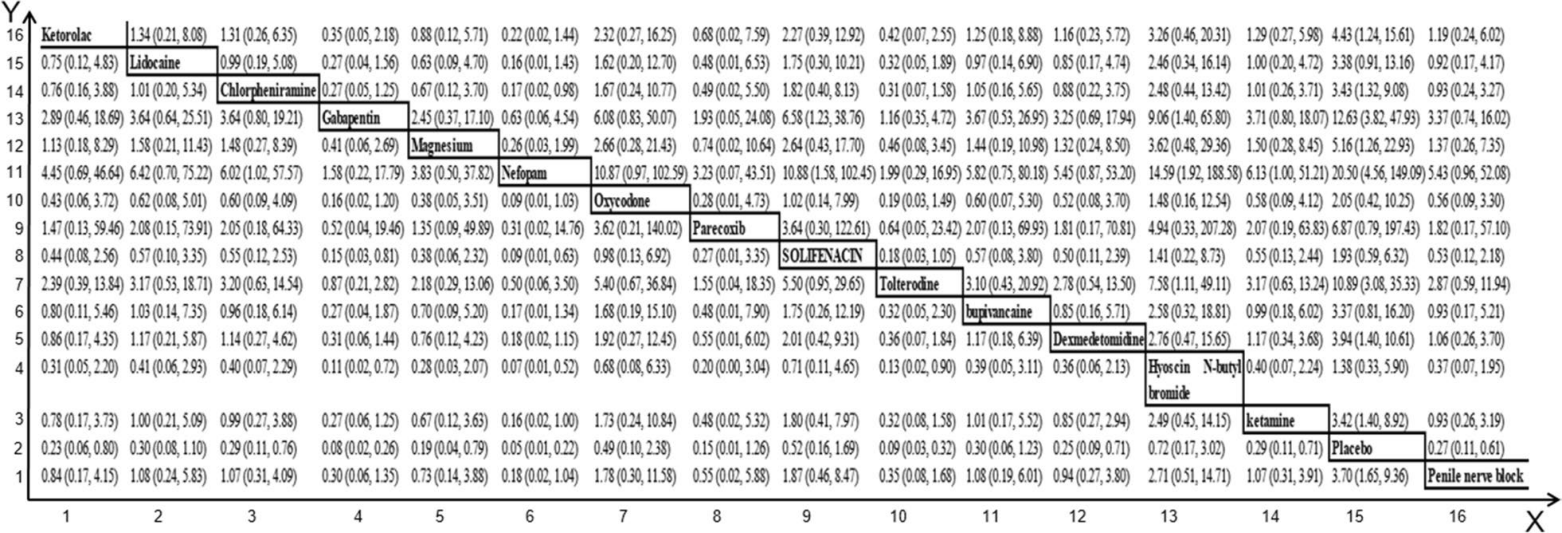
OR and 95%CI of network analysis index under consistency model.For example: Y15-X1 means “Ketorolac” relative to “Lidocaine”, OR is 0.75 and CI is (0.12, 4.83)

**Fig. 5:**
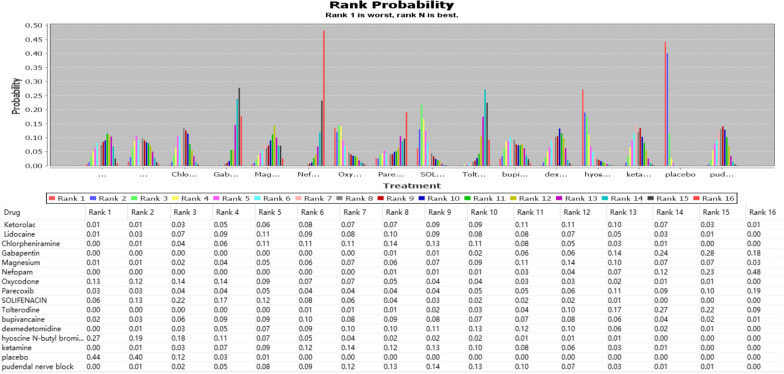
The rank of interventions for managing moderate to severe CRBD at 1 h after surgery. Rank 1 is the worst and Rank 16 is the best

**Fig. 6:**
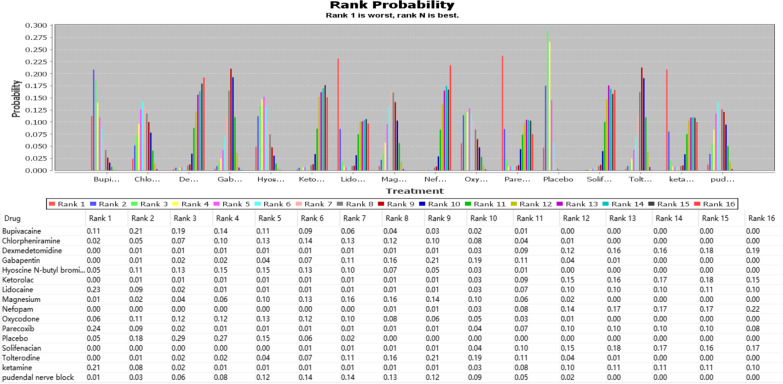
The rank of interventions for managing incidence of severe CRBD at 1 h after surgery. Rank 1 is the worst and Rank 16 is the best

Figure 4 shows the OR and 95%CI calculated by ADDIS for the analysis of the effect on the efficacy of the Bayesian model. Most interventions reduced the incidence of moderate to severe CRBD at 1 h after surgery to varying degrees, with nefopam having the most significant effect [OR:0.05 (0.01,0.22)]. Next, we ranked the comparative effectiveness of the interventions according to outcomes, which were calculated by using ADDIS (Figs. 5 and 6); Rank 16 showed the best results in both figures. Figures 5 and 6 also showed that Rank16, i.e., nefopam was the largest in included studies (0.48 and 0.22), thus suggesting it was the most effective in managing CRBD in terms of severity or reducing incidence.

The variance of inconsistency in the Bayesian model is calculated in Table 3. When the deviation between Random Effects Standard Deviation and Inconsistency Standard Deviation is close, the consistency of the Bayesian model is better. We observed low consistency in the present study, which was probably because of the few direct comparisons between interventions.

**Table 3:** The variance calculation of inconsistency in Bayesian model

Parameter	Median (95% Crl)
Random effects standard deviation	0.26 (0.01, 1.36)
Inconsistency standard deviation	0.94 (0.04, 2.39)

## Discussion

CRBD usually occurs after perioperative catheterization procedures; however, it is more common following urologic procedures, especially after transurethral resection of bladder tumors (TURBt) and transurethral resection of the prostate (TURP) [20]. Accordingly, 18 studies that met the inclusion criteria were included in this review, and the effects of 16 interventions were assessed. The major findings of our study are as follows: (1) nefopam was found to be the most effective in reducing the incidence of CRBD; (2) nefopam resulted as most effective in terms of managing the severity CRBD. Despite the lack of data, we reported our NMA because head-to-head comparisons of many of the investigated drugs for treating and preventing CRBD after urological surgery are still lacking.

Nefopam is non-opioid and non-steroidal derived from a non-sedative benzoxazocine and has been widely used in treating mild to moderate postoperative pain in different clinical settings [21, 22]. Its main mechanisms of analgesic effect include inhibition of serotonin, norepinephrine, and dopamine reuptake, as well as decreased activation of postsynaptic glutamate receptors by regulating calcium and sodium channels [23].

According to our results, nefopam resulted as most effective in managing CRBD as a combination of multiple mechanisms might contribute to the prevention of CRBD. First, this may be related to nefopam’s triple receptor reuptake inhibition. The raphe nucleus is the major source of serotonin-containing terminals in the spinal cord. The lumbosacral autonomic nucleus, also known as the motor nucleus of the sphincter, receives serotonergic input from the raphe nucleus, and stimulation of the raphe nucleus has been found to inhibit the bladder contractile reflex in cat and rat studies [24, 25]. As selective serotonin uptake inhibitors have inhibitory effects on an overactive bladder, we speculated that nefopam might also inhibit bladder activity by increasing serotonin in the central nervous system. Second, extracellular calcium influx through calcium channels is an important condition for activating the detrusor via muscarinic receptors and noradrenergic pathways [26], and the effects of nefopam on calcium channels may inhibit detrusor hyperactivity.

CRBD has aroused the large attention of urologists because of its higher incidence. In their study, Moataz et al*.* [27] established a descriptive epidemiological profile of patients with CRBD and determined its predictors. They concluded that improved surgical techniques could reduce the incidence of CRBD. Eun et al. reviewed managing CRBD, and concluded that a number of drugs, including gabapentin, solifenacin, tolterodine and so on, could improve CRBD after surgery. Nonetheless, most of these studies were conducted after the intubation of patients who were given the drug under anesthesia [28]. Currently, there are few studies on treating CRBD in patients with short-term or long-term catheterization of urinary system diseases and no prospective studies of such patients [28]. In a recent study, Marie et al. reviewed CRBD, describing the effectiveness of many drugs, including gabapentin, magnesium, dexmedetomidine, and ketamine. They suggested that perioperative drug therapy is beneficial for preventing postoperative CRBD; however, controversies remained about the rank of the effectiveness of various interventions [20]. Another NMA also analyzed the effect of some drugs, revealing that gabapentin 1200 mg ranked first in reducing the incidence of CRBD, while tolterodine was leading in terms of severity. This was inconsistent with our conclusion, which could be due to the following reasons: (1) the present analysis included more drugs and interventions; (2) included RCTs were published in the recent ten years; however, the former NMA did not include studies published in recent five years; (3) the technology of surgery and nursing has made great progress, which reduced the incidence of CRBD; (4) gabapentin acts by binding to the α2δ subunit of voltage-activated calcium channels; however, the exact mechanism of its action remains unclear., while it is known that nefopam manages CRBD through multiple mechanisms.

Our NMA comprehensively analyzed RCTs on CRBD published in the past decade, ranking almost all interventions and providing valuable references for managing CRBD. Nonetheless, there are still some limitations that should be considered. First, differences in patient characteristics and study designs in RCTs need to be considered when interpreting the outcomes. E.g., patients underwent different urological procedures, which led to marked differences in the incidence of postoperative CRBD and inevitable heterogeneity across included studies. Second, each drug was accompanied by side effects such as dizziness, nausea, vomiting, etc. However, we could not compare them in our NMA. Therefore, side effects should also be considered when choosing a therapeutic drug. Still, it is interesting that nefopam resulted as the most effective drug in our study, as well as the one that could manage pain after surgery, such as Laparoscopic Cholecystectomy and arthroscopic orthopedic surgery [29, 30] having a lower incidence of postoperative adverse effects in a recent study. Third, most studies were small RCTs, with more than half having an unclear or high risk of bias. The number of studies for each intervention was small. It was impossible to perform subgroup or sensitivity analyses due to the small number of studies. Fourth, a paucity of head-to-head comparisons between two interventions decreases the quality of network estimates by increasing loop-specific heterogeneity [31].

## Conclusion

Our data suggest that nefopam reduces the incidence of CRBD, improves the quality of life, and reduces serious adverse events. Although our study ranked these drugs, decisions regarding drug use will depend on clinical evaluation and patient discussion about the benefits and risks of prophylactic use of drugs. It should also be noted that the small number of studies on each intervention and heterogeneous patient limits our results. Thus, more high-quality RCTs are needed to perform head-to-head comparisons among different interventions to ensure NMA reliability.

## Supplementary Information

**Additional file 1**. Forest plot 1.

**Additional file 2**. Forest plot 2.

## Data Availability

The data and materials can be obtained by contacting the corresponding author.

## Abbreviations

CRBD: Catheter-related bladder discomfort
NMA: Network meta-analysis
ADDIS: Aggregate data drug inormation system
OR: Odds ratios
CI: Confidence intervals
RCT: Randomized controlled trials
PSRF: Potential Scale Reduced Factor
TURBt: Transurethral resection of bladder tumors
TURP: Transurethral resection of prostate
ULS: Urology laparoscopic surgery
UL: Ureteroscopic lithotripsy
PCNL: Percutaneous nephrolithotomy
