# Supplementary figures and images for: Comparative effectiveness of interventions for managing urological postoperative catheter-related bladder discomfort: a systematic review and network meta-analysis
Source: BMC Urol. 2023 Mar 3;23:29. doi: 10.1186/s12894-023-01195-9 (PMC9985303; doi:10.1186/s12894-023-01195-9)

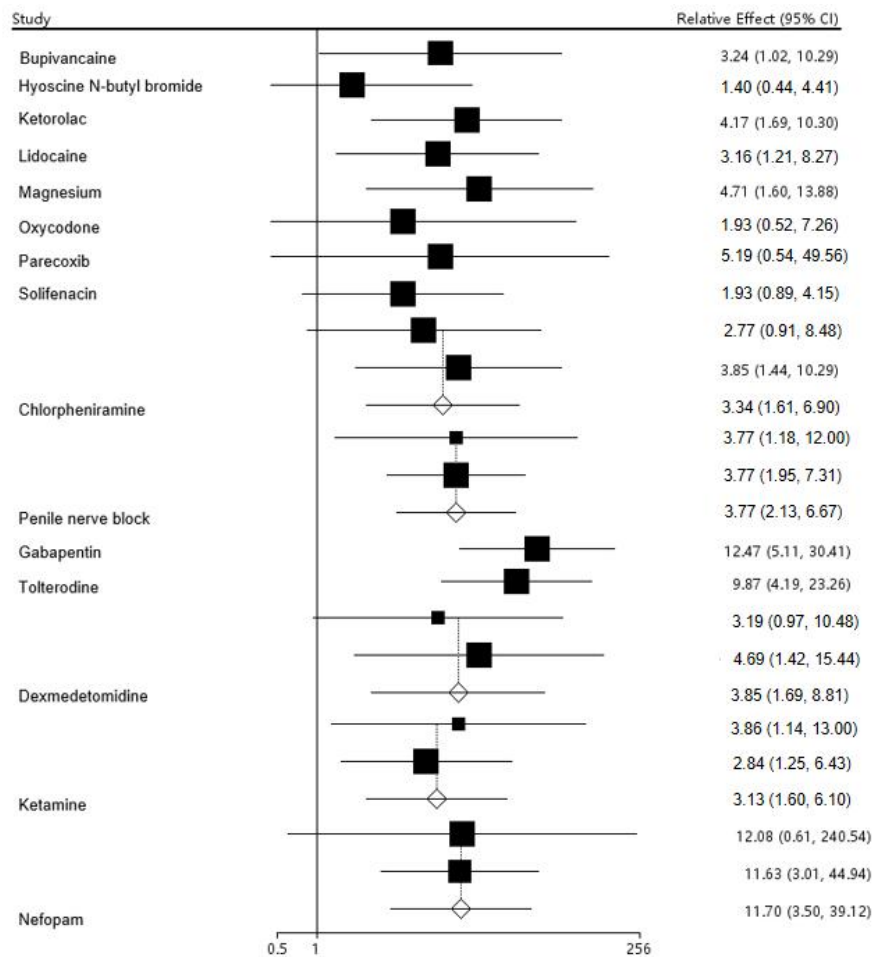

**Supplementary Fig.1** The forest plot shows OR and 95%CI of Interventions VS Placebo.

Supplement: Supplementary file 1 — Additional file 1. Forest plot 1. [file 12894_2023_1195_MOESM1_ESM.pdf]

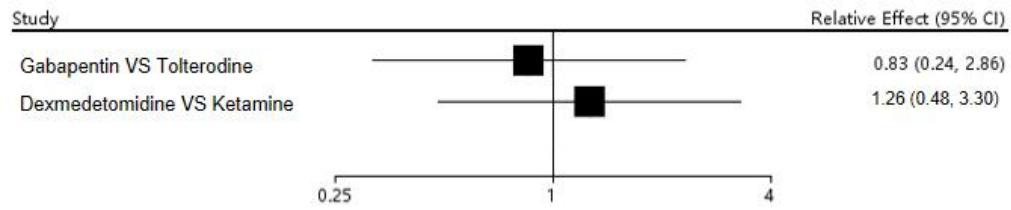

**Supplementary Fig.2** The forest plot shows OR and 95%CI of head-to-head comparisons of Interventions.

Supplement: Supplementary file 2 — Additional file 2. Forest plot 2. [file 12894_2023_1195_MOESM2_ESM.pdf]
